# Long-term follow-up of a randomized controlled trial on individualized fortification of human milk

**DOI:** 10.3389/fnut.2025.1669809

**Published:** 2026-01-07

**Authors:** Meiying Quan, Yu Zhang, Lejia Zhang, Chen Wang, Zhenghong Li

**Affiliations:** Department of Pediatrics, State Key Laboratory of Complex Severe and Rare Disease, Peking Union Medical College Hospital, Chinese Academy of Medical Science and Peking Union Medical College, Beijing, China

**Keywords:** PUMCH individualized fortification, preterm infants, physical growth, long-term follow-up, neurological development, metabolic diseases

## Abstract

**Background and aims:**

To evaluate short- and long-term outcomes of preterm infants from a previous RCT on individualized fortification (IF).

**Methods:**

Preterm infants from the Peking Union Medical College Hospital (PUMCH) IF study were enrolled. Data before age 2 were collected from medical records. Long-term follow-up was conducted at ages 6–10 years. Growth and development outcomes as well as biochemical indexes were compared between IF and standard fortification (SF) groups.

**Results:**

Fifty-one infants completed 2-year follow-up. The IF group showed significantly better outcomes in head circumference z-scores at CA 6 months (*p* = 0.035), hemoglobin at CA 12 months (*p* = 0.044), and weight *z*-scores at CA 24 months (*p* = 0.027). Twenty-five children completed long-term follow-up. No significant differences were found in lung function, cognitive scores, ferritin, hemoglobin, IGF, 25-OH-vitamin D, bone age, or metabolic parameters including BMI and lipid profile.

**Conclusion:**

PUMCH IF improved early growth outcomes, but no significant long-term health differences were observed.

## 1 Introduction

Nutritional support during early life plays a crucial role in the growth and development of preterm infants, influencing not only their physical growth but also their long-term neurological development and susceptibility to metabolic diseases (1, 2). Human milk (HM) is the preferred enteral nutrition for preterm infants, and fortifying it is essential for supporting the nutritional needs of these vulnerable infants. However, the suitability of a standard HM fortification for all preterm infants remains a subject of ongoing debate (2–4). Currently, there are three main strategies for HM fortification: standard fortification (SF), adjustable fortification, and targeted fortification. Despite these strategies, extrauterine growth retardation (EUGR) remains common in neonatal intensive care unit (NICU) (5).

The individualized fortification strategy at Peking Union Medical College Hospital (PUMCH) accounts for the unique nutritional composition of preterm human milk, as well as the specific metabolic and absorptive capacities of preterm infants. Fortification levels are determined based on the infant’s weight, the protein content of HM, and serum urea nitrogen levels, resulting in a five-tier stratification system. Levels 1, 2, and 3 represent super-fortification, in which the amount of fortifier is increased and additional protein powder is required beyond what is provided in standard commercial products. Level 0 corresponds to standard commercial fortification, whereas level −1 reflects reduced fortification with a lower amount of fortifier.

A previous randomized controlled trial (RCT) conducted at our center showed that, within the individualized fortification (IF) group, 62.5% (15/24) of preterm infants received HM fortified to level 1, 29.2% (7/24) to level 2, and 12.5% (3/24) to level 3. By the third week, the IF group exhibited a significantly higher weight gain velocity compared with the SF group (20.8 ± 7.9 vs. 14.9 ± 4.5 g/kg/day, *p* = 0.022). This IF strategy demonstrated a potential trend toward greater enteral protein intake and improved in-hospital growth. Notably, by week three, weight gain velocity in the IF group remained significantly superior to that of the SF group (6).

A recent study in Thailand supported these findings, reporting that infants in the adjusted fortification group achieved significantly greater weight gain (36.46 ± 6.09 vs. 25.78 ± 8.81 g/day; *p* = 0.001) and length gain (1.93 ± 0.57 vs. 1.12 ± 0.64 mm/day; *p* = 0.001) compared with those in the SF group. Moreover, protein intake was significantly correlated with both weight gain (*r* = 0.632, *p* < 0.001) and length gain (*r* = 0.577, *p* = 0.001) (7).

This study aims to follow up on preterm infants from the PUMCH individualized fortification trial to explore their short-term and long-term growth, neurological development, and the incidence of chronic diseases after hospital discharge.

## 2 Materials and methods

### 2.1 Study population

The study population consisted of preterm infants admitted to the NICU between September 2012 and August 2016, who had completed the PUMCH individualized fortification clinical trial. Inclusion criteria were as follows: preterm infants with a gestational age of less than 34 weeks, a birth weight between 800 and 1800 grams, and HM intake accounting for ≥80% of their enteral feeding volume during the RCT study period. Infants who had complete follow-up data were enrolled in the study, with long-term follow-up conducted between February 2023 and September 2023.

The IF group followed the protocols outlined in Tables 1, 2, while the SF group received commercially available standard fortification. Ethical approval for the study was granted by the PUMCH Ethics Committee (No. I23PJ501), and informed consent was obtained from the guardians of all participating infants.

**Table 1:** Individualized HM fortification strategy.

Weight (g)	Protein in breast milk (g/100 mL)	BUN (mmol/L)	Fortification level
<1,500	≥1.5	>5.0	Level 0
<1,500	≥1.5	3.2 ~ 5.0	Level 1
<1,500	≥1.5	<3.2	Level 2
<1,500	≤1.4	>5.0	Level 1
<1,500	≤1.4	3.2 ~ 5.0	Level 2
<1,500	≤1.4	<3.2	Level 3
≥1,500	≥1.5	>5.0	Level −1
≥1,500	≥1.5	3.2 ~ 5.0	Level 0
≥1,500	≥1.5	<3.2	Level 1
≥1,500	≤1.4	>5.0	Level 0
≥1,500	≤1.4	3.2 ~ 5.0	Level 1
≥1,500	≤1.4	<3.2	Level 2

BUN, blood urea nitrogen.

**Table 2:** Amounts of HM fortifiers and protein powder added at different fortification levels.

Fortification level	HMF (Packs/ 100 mL HM)	Protein powder (g/100 mL HM)	Supplemented protein by HMF and protein powder (g/100 mL HM)
Level −1	3	0	0.8
Level 0	4	0	1.1
Level 1	5	0	1.4
Level 2	5	0.4	1.8
Level 3	5	0.6	2

### 2.2 Study procedures

From February 2023 to September 2023, the preterm infants who participated in the initial trial were followed up, and outpatient data were analyzed. The IF strategy at PUMCH accounts for the unique nutritional composition of preterm human milk, as well as the specific metabolic and absorptive capacities of preterm infants.

**Figure 1:**
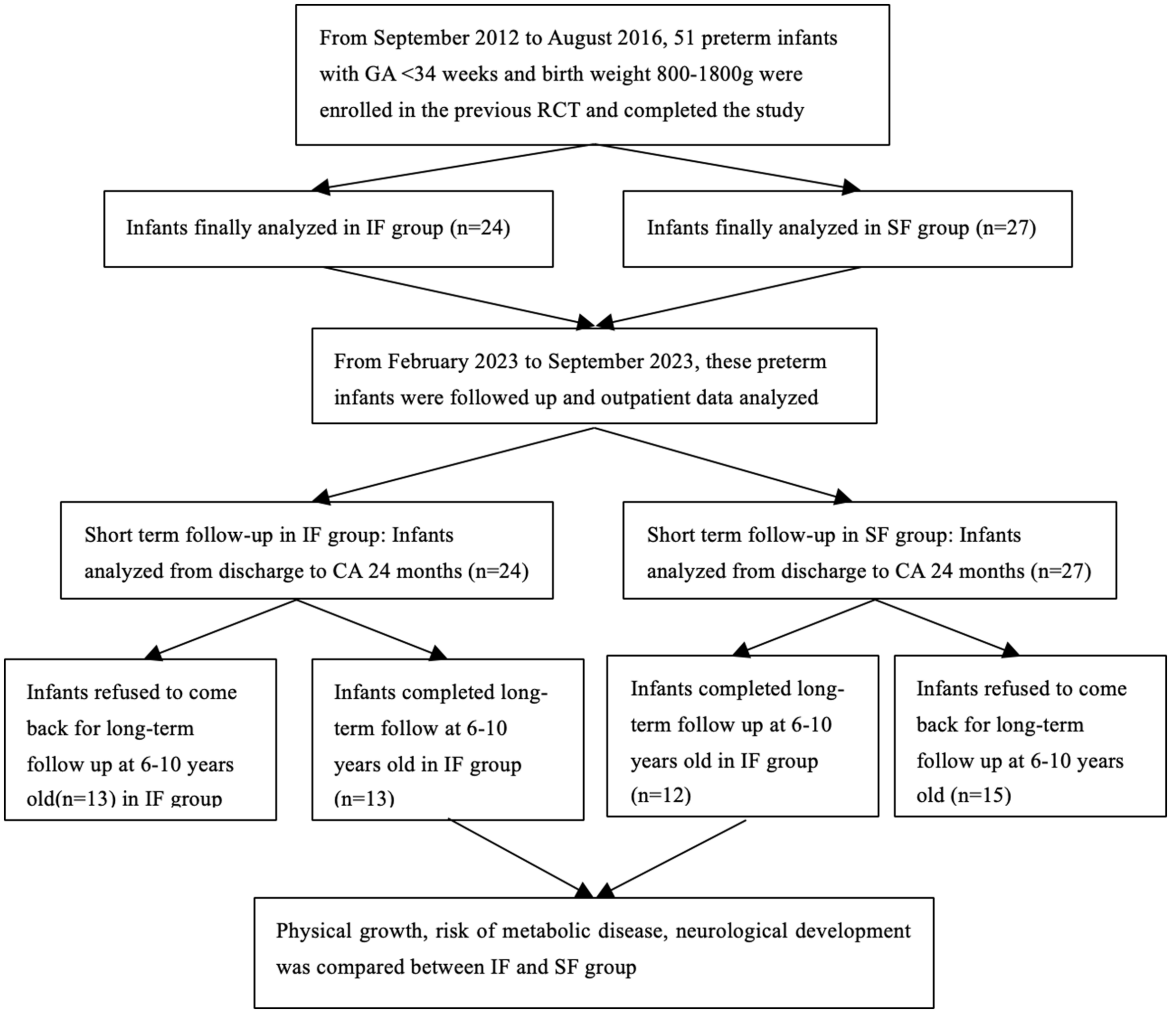
Participant flow chart. GA, gestational age; IF, individualized fortification; SF, standard fortification; CA, corrected age.

The participant flow chart is shown in Figure 1. The short-term follow-up period extended from discharge until the infants reached 24 months of corrected age (CA). Data were retrieved from the outpatient electronic medical records and included measurements of weight, length, and head circumference at CA of 40 weeks, 1 month, 3 months, 6 months, 9 months, 12 months, 18 months, and 24 months, as well as information on feeding and hemoglobin levels at birth as well as at CA of 6 months and 12 months. Weight, length, and head circumference were measured by trained nurses. Infants were placed on a calibrated, integrated weight-length measuring device without clothes, and length was measured from the top of the head to the heel with the infant fully extended. Head circumference was measured with a soft tape around the head, from the upper edge of the eyebrow arches to the occipital prominence.

For preterm infants before reaching 40 weeks of CA, physical growth was assessed using the intrauterine growth curve for preterm infants (2013 Fenton curve). After reaching 40 weeks CA, the World Health Organization (WHO) growth charts were used to calculate the weight, length, and head circumference *Z*-scores (8–10). The *Z*-scores were calculated as follows: Weight *Z*-score = (measured weight-average weight for that gestational age)/standard deviation of weight for that gestational age. Length *Z*-score = (measured length-average length for that gestational age)/standard deviation of length for that gestational age. Head circumference *Z*-score = (measured head circumference-average head circumference for that gestational age)/standard deviation of head circumference for that gestational age.

The long-term follow-up was conducted between ages 6 and 10 years. During this period, families were contacted by phone and invited to return to the outpatient clinic. Physical development assessments included weight (kg) and height (cm), alongside evaluations for respiratory, digestive, neurological, endocrine, and allergic conditions. Biochemical indexes included cholesterol (mmol/L), triglycerides (mmol/L), hemoglobin (g/L), ferritin (μg/mL), 25-hydroxyvitamin D (ng/mL), IGF-1 (ng/mL), and HbA1c (%). Additionally, bone age, lung function (FEV1, FEV1/FVC), and intellectual development were assessed. Cognitive function was evaluated using the Chinese Wechsler Primary Scale of Intelligence with Language IQ covering general knowledge, vocabulary, arithmetic, similarities, and comprehension, and the Performance IQ including animal house, picture completion, maze, geometric patterns, and block design. Total IQ score was calculated by combining Language and Performance IQ scores according to the corresponding conversion tables (11).

### 2.3 Statistical analysis

Statistical analysis was performed using SPSS version 26.0. Continuous variables with a normal distribution were compared using independent sample t-tests, while non-normally distributed data were analyzed with the Mann–Whitney U test. Categorical variables were compared using the chi-square test, adjusted chi-square test, or Fisher’s exact test, as appropriate. A *p*-value of less than 0.05 was considered statistically significant.

## 3 Results

### 3.1 Short-term follow-up

A total of 51 preterm infants completed the PUMCH IF trial during their NICU hospitalization. The follow-up rate for infants under 2 years of age was 100%. Z-scores for weight, length, and head circumference at CA of 40 weeks, 1, 3, 6, 9, 12, 18, and 24 months, as well as hemoglobin levels at discharge, CA of 6 and 12 months, were compared between groups. Statistically significant differences were observed in head circumference at CA 6 months, weight at CA 24 months, and hemoglobin levels at CA 12 months with the IF group showing superior outcomes compared with the SF group (see Table 3).

**Table 3:** Comparison of short-term follow-up outcomes.

Grouping	GA	Weight *Z*-score at CA 40W	Length *Z*-score at CA 40W	HC *Z*-score at CA 40W	Weight *Z*-score at CA 1M	Length *Z*-score at CA 1M	HC *Z*-score at CA 1M	Weight *Z*-score at CA 3M	length *Z*-score at CA 3M	HC *Z*-score at CA 3M
SF group (*n =* 27)	30.4 ± 1.2	0.1 ± 1.2	−0.4 ± 0.8	0.1 ± 0.8	1.0 ± 1.2	0.8 ± 1.3	1.0 ± 1.1	1.1 ± 1.2	0.9 ± 1.2	0.9 ± 0.8
IF group (*n =* 24)	30.4 ± 1.8	0.2 ± 0.8	−0.2 ± 0.9	0.2 ± 0.8	1.2 ± 1.4	0.5 ± 1.7	1.0 ± 1.4	1.3 ± 1.1	1.0 ± 1.3	1.1 ± 0.9
Statistical value	−0.119	−0.202	−0.345	−0.492	−0.471	0.584	0.012	−0.591	−0.331	−1.02
*p* value	0.906	0.841	0.732	0.626	0.64	0.562	0.991	0.558	0.461	0.314

Grouping	Weight *Z*-score at CA 6M	Length *Z*-score at CA 6M	HC *Z*-score at CA 6M	Weight *Z*-score at CA 9M	Length *Z*-score at CA 9M	HC *Z*-score at CA 9M	Weight *Z*-score at CA 12M	Length *Z*-score at CA 12M	HC *Z*-score at CA 12M
SF Group (*n* = 27)	0.7 ± 1.2	0.6 ± 1.1	0.5 ± 0.8	0.5 ± 1.1	0.7 ± 1.1	0.5 ± 0.8	0.2 ± 1.0	0.5 ± 1.2	0.3 ± 1.0
IF Group (*n* = 24)	1.1 ± 1.0	1.1 ± 1.4	1.2 ± 1.0	0.7 ± 1.1	0.7 ± 0.9	0.7 ± 0.8	0.8 ± 1.1	0.6 ± 1.2	0.6 ± 1.2
Statistical value	−1.173	−1.186	−2.202	−0.382	−0.136	−0.635	−1.539	−0.208	−0.914
*p* value	0.249	0.244	0.035	0.706	0.893	0.531	0.136	0.837	0.37

Grouping	Weight *Z*-score at CA 18M	Length *Z*-score at CA 18M	HC *Z*-score at CA 18M	Weight Z-score at CA 24M	Length *Z*-score at CA 24M	HC *Z*-score at CA 24M	Hb at birth	Hb at CA 6M	Hb at CA 12M
SF group (*n* = 27)	−0.1 ± 0.9	0.2 ± 1.0	0.2 ± 0.9	0.03 ± 0.62	0.2 ± 1.4	0.0 ± 0.6	191.9 ± 26.5	116.9 ± 11.7	111.3 ± 13.2
IF group (*n* = 24)	0.7 ± 0.8	0.5 ± 0.8	0.7 ± 0.8	1.08 ± 0.77	0.5 ± 0.7	0.7 ± 0.9	186.9 ± 29.3	121.8 ± 8.8	126.0 ± 9.4
Statistical value	−2.021	−0.788	−1.313	−2.587	−0.461	−1.368	0.618	−1.094	−2.268
*p* value	0.061	0.443	0.21	0.027	0.654	0.214	0.54	0.287	0.044

GA, gestational age; SF, Standard Fortification; IF, Individualized Fortification; HC, head circumference; W, weeks; M, months.

### 3.2 Long-term follow-up

A total of 25 preterm infants (13 from the IF group and 12 from the SF group) were successfully recruited for long-term follow-up. The mean age at follow-up was 8.6 ± 1.3 years in the IF group and 8.7 ± 1.2 years in the SF group (*p* = 0.796). No significant differences were observed between the groups in *Z*-scores for height, weight, or BMI, nor in measures of insulin-like growth factor, blood cholesterol, blood triglycerides, HbA1c, 25-hydroxyvitamin D, bone age, lung function, or intellectual development (see Table 4).

**Table 4:** Comparison of long-term follow-up outcomes.

Grouping	Follow-up age (years)	Weight Z-score	Length Z-score	BMI (kg/cm^2^)	Bone age (years)	25(OH)VD (ng/mL)	Wechsler Scale (points)	Allergic diseases	Abnormal lung function
SF group (*n* = 12)	8.7 ± 1.2	−0.08 ± 1.3	0.18 ± 1.12	16.1 ± 2.9	8.4 ± 2.0	20.9 ± 7.8	121.4 ± 16.7	25%	25%
IF group (*n* = 13)	8.6 ± 1.3	0.02 ± 0.99	0.41 ± 0.71	16.3 ± 3.6	7.6 ± 1.5	24.4 ± 9.9	126.6 ± 15.8	7.70%	38%
Statistical value	0.262	0.268	0.588	−0.167	1.072	−0.984	−0.799	1.391	0.52
*p* value	0.796	0.817	0.562	0.869	0.295	0.336	0.432	0.322	0.673

Grouping	FEV1 (%)	FEV1/FVC (%)	Ferritin (μg/mL)	Hemoglobin (g/L)	IGF1 (ng/mL)	Glycated hemoglobin (%)	Cholesterol (mmol/L)	Triglycerides (mmol/L)
SF group (*n* = 12)	94.0 ± 6.9	96.9 ± 4.3	50.8 ± 11.6	134.7 ± 4.7	254.1 ± 104.2	5.2 ± 0.13	4.17 ± 0.4	0.96 ± 0.4
IF group (*n* = 13)	98.6 ± 12.3	99.4 ± 4.0	55.4 ± 30.3	131.8 ± 11.8	228.1 ± 106.8	5.2 ± 0.22	4.36 ± 0.4	0.88 ± 0.4
Statistical value	1.151	1.493	−0.49	0.78	0.616	0.502	−1.154	0.48
*p* value	0.264	0.148	0.632	0.448	0.544	0.622	0.265	0.636

BMI, Body Mass Index; 25(OH)VD: 25-Hydroxyvitamin D, IGF1: Insulin-like Growth Factor.

## 4 Discussion

### 4.1 The role of individualized fortification in the growth of preterm infants

IF is customized to the changing composition of HM over time, thereby meeting the evolving nutritional needs of preterm infants (4). Our previous RCT demonstrated that preterm infants in the IF group achieved significantly higher weight gain velocity by the third week compared with the SF group, an effect closely linked to the cumulative benefit of increased protein intake from enteral nutrition (6). In the present follow-up study, we further evaluated both the short-term and long-term outcomes.

During the short-term follow-up, IF effectively met the nutritional needs of preterm infants and significantly improved Z-scores for head circumference at CA 6 months and weight at CA 24 months, highlighting its critical role in early developmental outcomes. Beyond promoting growth, IF was also associated with higher hemoglobin levels at CA 12 months. As widely recognized, iron supplementation during the initial weeks after birth is essential for preventing anemia (12). Fortified HM provides preterm infants with adequate iron during this critical period, supporting hemoglobin synthesis and reducing the risk of anemia. In this regard, the IF group demonstrated clear advantages over the SF group.

When evaluating the long-term effects of IF, special attention was given to the metabolic health of infants following early nutritional fortification. Several studies have indicated that early nutritional interventions can have lasting impacts throughout childhood, affecting metabolic parameters such as BMI, body fat percentage, and insulin sensitivity (13). Theoretically, HM fortification could lead to faster fat accumulation in preterm infants and accelerated growth. Specifically, early-life exposure to higher protein intake, such as that provided by fortified human milk, may stimulate increased insulin and IGF-1 activity, driving rapid weight gain and a disproportionate increase in adipose tissue. This pattern of early accelerated growth is thought to heighten the likelihood of an earlier and more pronounced adiposity rebound, which is a well-established predictor of later childhood and adult obesity (14). In our long-term follow-up, however, no significant differences were observed between the IF and SF groups in height, weight, Z-scores, BMI, or insulin-like growth factor. Similarly, cholesterol and triglyceride levels did not differ significantly, and neither group showed signs of metabolic syndrome, and neither group showed signs of metabolic syndrome. These findings likely reflect the multifactorial nature of long-term development, in which later environmental influences, dietary patterns, disease exposure, and socioeconomic factors may attenuate or obscure the impact of early nutritional interventions.

### 4.2 Individualized fortification and bone health in preterm infants

Vitamin D is an essential nutrient for maintaining bone health, playing a key role in calcium and phosphorus absorption, bone mineralization, and skeletal growth. By enhancing intestinal absorption of calcium and phosphorus, vitamin D helps maintain adequate blood calcium levels, supports bone mineralization, and contributes to overall bone strength. Moreover, vitamin D regulates the activity of osteoblasts and osteoclasts, thereby influencing bone remodeling and promoting appropriate bone mass accrual during growth (15). A Dutch study reported that fat intake (g/kg/day) during the first 4 weeks of life was positively associated with bone mineral density at corrected full term. Furthermore, at CA 6 months, both early-life protein and fat intake (g/kg/day) were independent predictors of bone mineral density. Notably, fat intake during the first 4 weeks was significantly correlated with bone density at CA 6 months (16). These findings indicate that early nutritional fortification and adequate nutritional support during infancy are crucial for optimal bone health in the first few months of life.

In our study, no significant differences in bone health were observed between the IF and SF groups, and all measures remained within the normal range. These findings indicate that bone health in preterm infants is shaped by multiple factors. With appropriate nutritional interventions and regular monitoring, normal bone development can be promoted and the risk of bone-related complications effectively reduced (17).

### 4.3 Individualized fortification and neurological development

The IF strategy increased protein intake for extremely preterm infants, with super-fortification levels advancing from level 1 to level 3. Protein is a critical nutrient for brain development, supporting neuronal proliferation and differentiation (3). The effect of IF on intellectual development may therefore depend on the provision of optimized protein during early life. Notably, head circumference, a key indicator of brain volume, was improved with IF of HM, suggesting that early intervention through IF creates favorable conditions for brain growth and development (18).

In a similar vein, a study from in Taiwan showed that the early administration of high-concentration liquid HM fortifiers in extremely low birth weight preterm infants significantly improved short-term growth and development. Infants receiving the high-concentration fortifier achieved markedly higher daily HM intake during the first 4 weeks after birth. Notably, the benefits extended into early childhood, at a two-year follow-up, the fortified group demonstrated significant gains in growth and neurodevelopment. At 24 months of age, infants in the high-concentration group had significantly higher language (*p* = 0.048) and motor (*p* = 0.032) scores compared with controls (19).

In the long-term follow-up, intellectual development was assessed using the Wechsler Intelligence Scale with no statistically significant difference between the two groups (*p* = 0.432). Despite observing greater protein intake and improved head circumference growth during early infancy in the IF group, these advantages did not persist into school age, nor did they translate into measurable differences in cognitive outcomes. Several mechanisms may underlie this long-term convergence. First, cognitive development is highly sensitive to a broad range of post-discharge influences—including the home learning environment, caregiver educational level, language exposure, and access to early childhood education. These environmental and socioeconomic factors often exert a much stronger effect on school-age cognition than early nutritional differences alone, and may therefore dilute or overshadow subtle early-life nutritional advantages. Second, socioeconomic disparities can shape developmental trajectories through differences in nutrition quality after hospital discharge, healthcare utilization, parental responsiveness, and opportunities for cognitive stimulation. Even small variations in these factors across families may contribute to the attenuation of early group differences. Third, neurodevelopmental plasticity in the first years of life may enable compensatory processes in infants who experience suboptimal early growth. As both groups ultimately achieved adequate growth by later infancy and received routine standardized follow-up care, the window of vulnerability may have narrowed, leading to equivalent cognitive outcomes at school age (20). Nonetheless, PUMCH IF, through optimized early nutritional support, may provide a biological basis for improved cognitive development. Early improvements in head circumference and hemoglobin levels could serve as favorable early indicators. Further investigation into cognitive and behavioral outcomes during adolescence and adulthood, along with a comprehensive evaluation of the long-term neurological effects of IF, is warranted.

### 4.4 Individualized fortification and pulmonary function

Studies have demonstrated that compared with full-term infants, preterm infants exhibit reduced pulmonary function during school age. More than 50% of infants born before 25 weeks of GA show abnormal lung capacity, most notably characterized by obstructive ventilatory dysfunction. Both FEV1 and the FEV1/FVC ratio are significantly reduced in this population, and these infants are at an increased risk for asthma diagnosis. Early nutritional support plays a crucial role in lung development, as optimal nutritional status supports alveolar formation, airway development, and reduces the incidence of bronchopulmonary dysplasia (21).

HM is rich in immune factors, such as immunoglobulin A and lactoferrin, which not only enhance immune defense but also protect lung function indirectly by reducing the risk of pulmonary infection (22). A previous study specifically explored the relationship between early nutrition and pulmonary function in preterm infants. In a 6-year follow-up, extremely preterm infants discharged on HM were randomly assigned to receive either fortified or unfortified HM, while non-breastfed infants received preterm formula until reaching a CA of 4 months. Lung function tests, including exhaled nitric oxide, airway resistance, and bronchodilator response, were conducted at age 6. The results indicated that protein-fortified nutrition after discharge improved lung function in extremely preterm infants (23).

In the long-term follow-up of this study, pulmonary function was evaluated in both the PUMCH IF and SF groups, and no significant differences were detected. This finding may be related to the relatively short follow-up duration and the presence of multiple confounding factors. The follow-up period in this study spanned ages 6 to 10, whereas full maturation of pulmonary function and the manifestation of long-term effects may require a longer observation window. Moreover, pulmonary outcomes are shaped not only by early nutritional status but also by a variety of later-life influences, including exposure to environmental pollutants, lifestyle habits, and levels of physical activity. Thus, individualized HM fortification may be only one of several determinants of pulmonary function, with other factors likely exerting substantial effects during the follow-up period.

### 4.5 Features and potential value of PUMCH individualized fortification

A defining feature of the PUMCH IF strategy is its ability to precisely adjust nutrient intake through stratified fortification, thereby accommodating the diverse growth demands of preterm infants. By increasing the provision of protein and other key nutrients, this approach supports improvements in both weight gain and head circumference growth. Compared with SF, the individualized model offers greater flexibility and responsiveness, particularly for extremely low birth weight infants and those experiencing rapid growth. Optimizing weight and head circumference during these critical early developmental periods may yield meaningful long-term benefits for neurological maturation and immune function (24).

## 5 Conclusion

Our findings suggest that PUMCH IF improves short-term protein intake and early head circumference growth in preterm infants without increasing feeding intolerance or adverse events. Although these early advantages did not translate into measurable differences in school-age cognitive outcomes, the approach remains safe and physiologically sound. Based on the available evidence, clinicians may consider using individualized fortification—particularly in very preterm or growth-restricted infants—to help close early nutritional gaps and support optimal early growth trajectories.

At the same time, the absence of long-term neurodevelopmental differences highlights the essential importance of post-discharge nutritional monitoring, family support, and early developmental interventions, which may exert a larger influence on long-term cognitive outcomes than in-hospital nutrient optimization alone. Therefore, IF should be viewed as one component of a continuous, development-centered nutritional strategy extending from the NICU to early childhood. Larger, adequately powered trials with long-term follow-up are still needed to determine which subgroups benefit most and to refine implementation in routine practice.

## Data Availability

The raw data supporting the conclusions of this article will be made available by the authors, without undue reservation.
